# Exploring risk groups for concomitant injuries in facial fracture patients: a data-driven analysis

**DOI:** 10.1007/s00068-026-03342-x

**Published:** 2026-09-29

**Authors:** Roope Mustasilta, Elias Kultanen, Tero Puolakkainen, Aleksi Haapanen, Hanna Thorén, Johanna Snäll

**Affiliations:** 1 Department of Oral and Maxillofacial Diseases, University of Helsinki and Helsinki University Hospital, Helsinki, Finland; 2 Department of Oral and Maxillofacial Diseases, Central Finland Central Hospital Nova, Jyväskylä, Finland; 3 Department of Oral and Maxillofacial Surgery, Department of Oral and Maxillofacial Diseases, University of Turku, Turku University Hospital, Turku, Finland

**Keywords:** Face, Bone fracture, Facial injuries, Jaw fractures, Injuries, Multiple trauma

## Abstract

**Purpose:**

Patients with facial fractures are a diverse group with varying injury mechanisms and extent of injury. These patients often suffer from concomitant injuries (CIs) of different severity outside the facial region, some of which are life-threatening and may require urgent care. Therefore, early identification of patients at risk for CI is important. This study aimed to investigate exploratory risk groups for concomitant injuries in patients with facial fractures, focusing on the impact of injury energy, sex and age on the prevalence and type of concomitant injury.

**Methods:**

In this retrospective cohort study, a data-driven analysis was used to perform statistical analysis and identify risk groups for different CIs. The study included 4170 patients with facial fractures evaluated at tertiary trauma centres from January 2013 to October 2020.

**Results:**

70.9% of patients with facial fractures were male. Patients’ mean age was 45.7 years. Of the injury mechanisms, 36.0% were classified as high-energy trauma. In all, 31.7% of facial fracture patients had some type of CI. Female patients had more CIs than male patients (34.0% vs. 30.8%). The occurrence of CIs increased progressively with age, peaking in the age group 90–94 years, where over 55% of patients experienced CIs. However, considerable variation was observed in CI types across age groups.

**Conclusion:**

Due to the high CI risk in facial fracture patients, in conjunction with assessment and imaging of the facial trauma, we encourage routinely ruling out CIs, with a special focus on high-risk CI groups.

**Supplementary Information:**

The online version contains supplementary material available at https://doi.org/10.1007/s00068-026-03342-x.

## Introduction

Patients presenting with facial fractures are a wide and diverse group with a range of injuries in the facial region [1–5]. Facial fracture patients often also sustain concomitant injuries (CIs) to parts of the body beyond the facial region.

The severity of CIs varies, and these injuries may require more urgent diagnosis and treatment than the facial fracture(s) [5]. These injuries range from central nervous system, vascular, spinal, thoracic, abdominal, and pelvic injuries to extremity injuries, with brain and extremity injuries being the most frequently reported [1, 3–7].

The occurrence of CIs in the facial fracture population has been reported to range from 18% to 35% [1, 3, 6–9]. In patients with panfacial fractures, the prevalence of CI has been found to be as high as 53% [2]. Increased risk of CI is influenced by numerous factors, from patient characteristics to energy or mechanism of injury [1–5, 7, 9, 10]. In addition, despite their considerable prevalence, CIs may go undetected [11–13].

Our recent studies, using conventional statistical methods, have highlighted the risks of CIs, particularly in elderly patients and in high-energy injuries [3–5, 7]. To further investigate and detect specific risk groups in a larger dataset, we utilized data-driven analysis for CI risk group identification. Our hypothesis was that by data-driven modelling we could identify CI risk groups that have remained previously undetected in conventional analyses of facial fracture patients.

## Materials and methods

### Study design

This retrospective cohort study included data of facial fracture patients evaluated in the tertiary trauma centres of Töölö and Children’s Hospital, Helsinki University Hospital, Helsinki, Finland from January 2013 to October 2020.

### Ethics approval

The study was approved by Helsinki University Hospital research board. According to Finnish legislation on medical research, using public and published data, registry and documentary data, and archive data do not require ethical board processing. Due to the retrospective nature of the study, the Helsinki University Hospital research board waived the need of obtaining informed consent. All methods were carried out in accordance with the Declaration of Helsinki.

### Inclusion and exclusion criteria

Data were retrieved from electronic patient records according to ICD-code for any craniofacial fracture (S02*), and the patient cases were manually reviewed. Patients with radiologically confirmed facial fractures were included in the analyses, while patients with isolated skull fractures occurring outside the facial thirds were excluded from the study.

### Study variables

The primary outcome variable was the presence of CI. The secondary outcome variable was the type of CI. The predictor variables were age, sex, and high-energy injury. Additional analyses were conducted for subgroups of assaults and ground-level falls. In addition, the number of CIs and the mortality rate during hospitalization were reported.

CIs were defined as injuries in other regions of the body, excluding facial bones. CIs were classified into the following categories based on radiological confirmation: (1) traumatic brain injury, (2) skull fracture (excluding fractures of the facial third), (3) blunt cerebrovascular injury, (4) cervical spine injury, (5) upper limb injury, (6) lower limb injury, (7) thoracic injury, (8) non-cervical spine injury, and (9) abdominal injury. Minor soft-tissue injuries and brain concussions without radiological confirmation of traumatic brain injury were excluded as CIs.

The mechanism of injury was classified as assault, falling on ground level, falling from a height (including falling from stairs), traffic accidents (including bicycle and e-scooter accidents), other accidents (e.g. rollerblading, kick scootering, skateboarding, downhill skiing, and other types of sport accidents), and being struck by an object.

Injury energy was divided into high-energy injury and low-energy injury. The classification for high-energy and low-energy injury was done based on earlier studies [14]. High-energy injuries were caused by injury mechanisms with high levels of force such as traffic accidents and falls from height. In contrast, low-energy injuries were caused by injury mechanisms with significantly lower levels of force such as ground-level falls and assaults.

In addition, the types of facial fractures were reported and classified as exclusively mandibular, exclusively midface, exclusively upper face, and combination fractures of the facial thirds.

### Data analysis

All statistical analyses were performed using Python (version 3.14.2) within a Jupyter Notebook environment, managed via Anaconda Distribution. The patient cohort was first stratified into low-energy and high-energy injury groups. The analysis proceeded in two stages, utilizing the pandas library for data manipulation. Initially, a hypothesis-driven approach assessed risk in predefined elderly populations (70+, 80+, 90 + years) by calculating conditional probabilities and 95% Wilson confidence intervals via the statsmodels library. Subsequently, a data-driven discovery phase was employed for each specific injury; machine learning-based decision tree classifiers, implemented with scikit-learn, were used to identify the most significant predictors and their critical thresholds. A detailed description of the parameters used for creating the data-driven model is provided in Supplementary material file 1. The model evaluated all candidate split thresholds and selected a 1.2-fold probability threshold based on the Gini impurity criterion, as this threshold provided the greatest reduction in node impurity. Results were reported only for groups with at least 10 cases. To evaluate the relative importance of injury kinetics, age and sex were used as predictors. Finally, the high-risk profiles identified by the trees were quantitatively validated by calculating their precise conditional probabilities and 95% Wilson confidence intervals.

## Results

Descriptive statistics of 4170 facial fracture patients are presented in Table 1. Of these patients, 2957 (70.9%) were male. The mean age of the study population was 45.7 years (median 42.4 years, range from 0.5 to 102.5 years). The most common fracture type was exclusively midfacial fracture (46.9%).

**Table 1:** Descriptive statistics of 4170 facial fracture patients

Variable	Number of patients	%
Total	4170	100
**Sex**		
Male	2957	70.9
Female	1213	29.1
**Age (yr)**		
Mean	45.7	
Median	42.4	
Range	0.5–102.5	
**High-energy injury**		
Yes	1500	36.0
**Fracture type**		
Exclusively midface	1954	46.9
Exclusively mandible	1195	28.7
Upperface	128	3.1
Combined	893	21.4
**Concomitant injury (CI)**		
Yes	1322	31.7
**Type of concomitant injury**		
Traumatic brain injury	598	14.3
Skull fracture	428	10.3
Blunt cerebrovascular injury	70	1.7
Cervical spine injury	121	2.9
Upper limb injury	422	10.1
Lower limb injury	146	3.5
Thoracic injury	341	8.2
Non-cervical spine injury	106	2.5
Abdominal injury	61	1.5
**Mortality**	55	1.3

Overall, the most frequent mechanism of injury was fall at ground level (31.8%), followed by assault (27.6%) and traffic accident (20.1%). Of injuries, 36.0% were defined as high-energy based on the description of the injury event (Table 2). The most frequent high-energy mechanism of injury was traffic accident (55.9%), followed by fall from height (28.3%). In contrast, the most frequent low-energy mechanism of injury was fall at ground level (49.7%), followed by assault (38.2%).

**Table 2:** Mechanism of injury and classification into high-energy injury and low-energy injury

Variable	All patients	%	High-energy injury	%	Low-energy injury	%
Total	4170	100	1500	100	2670	100
**Mechanism of injury**						
Fall at ground level	1328	31.8	0	0	1328	49.7
Assault	1153	27.6	132	8.8	1021	38.2
Traffic accident	839	20.1	839	55.9	0	0
Fall from stairs or height	429	10.3	424	28.3	5	0.2
Struck or hit by object	359	8.6	67	4.5	292	10.9
Other	62	1.5	38	2.5	24	0.9
**Concomitant injury (CI)**						
Yes	1322	31.7	862	57.5	460	17.2

In all, 31.7% of facial fracture patients had some type of CI (Table 2). In high-energy injuries, 57.5% of facial fracture patients sustained at least one CI. The corresponding proportion for low-energy injuries was 17.2% (Table 2). The most common CI was traumatic brain injury (14.3%), followed by skull fracture (10.3%) and upper limb injury (10.1%). The least frequent CI was abdominal injury (1.5%). The mortality rate during hospitalization was 1.3% (Table 1).

**Fig. 1:**
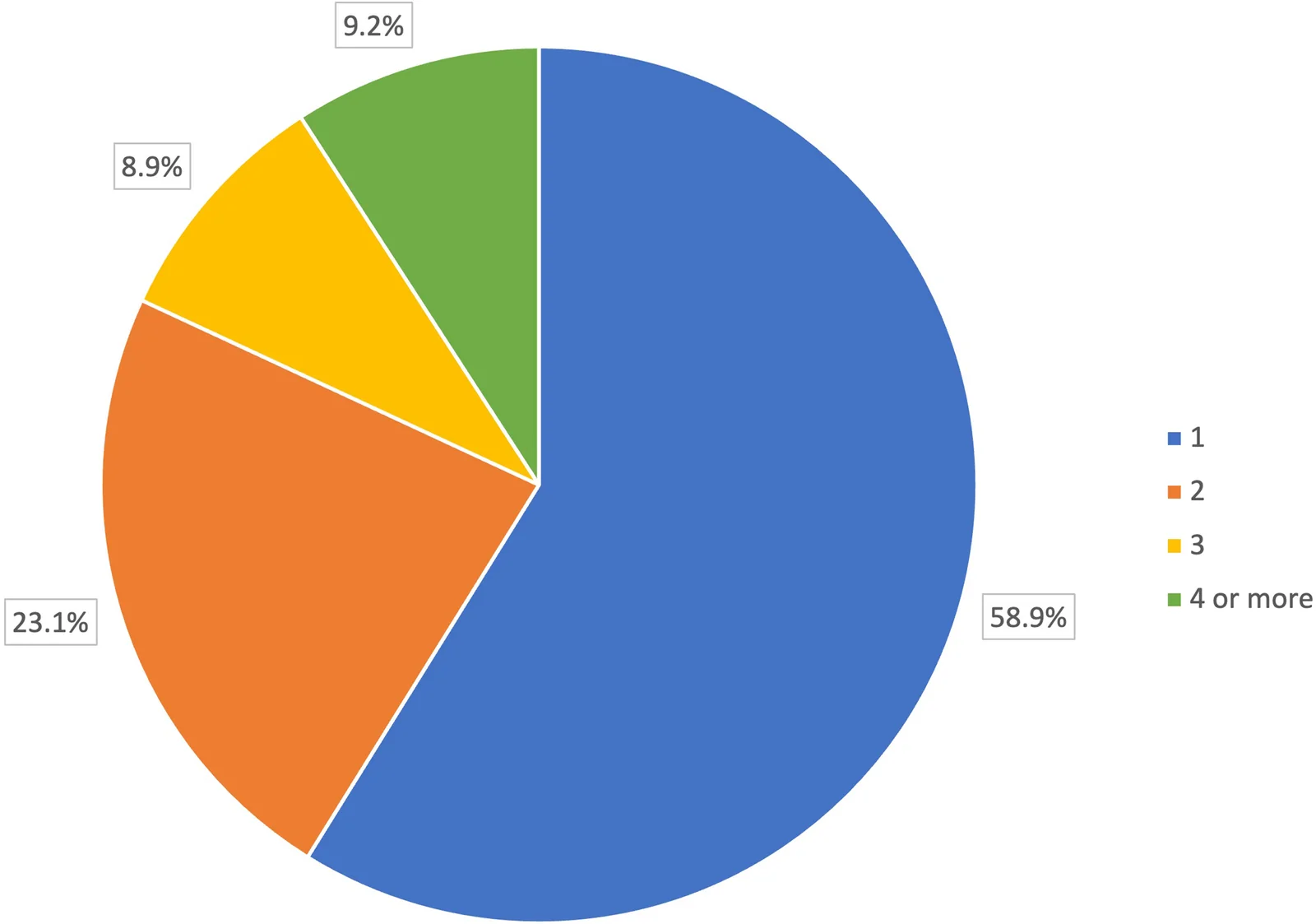
Number of concomitant injuries in facial fracture patients. Mean 1.7, median 1, range 1–7

The number of CIs varied (Fig. 1). In patients with facial fractures and CIs, 58.9% had one CI. Altogether 9.2% of patients had four or more CIs.

**Fig. 2:**
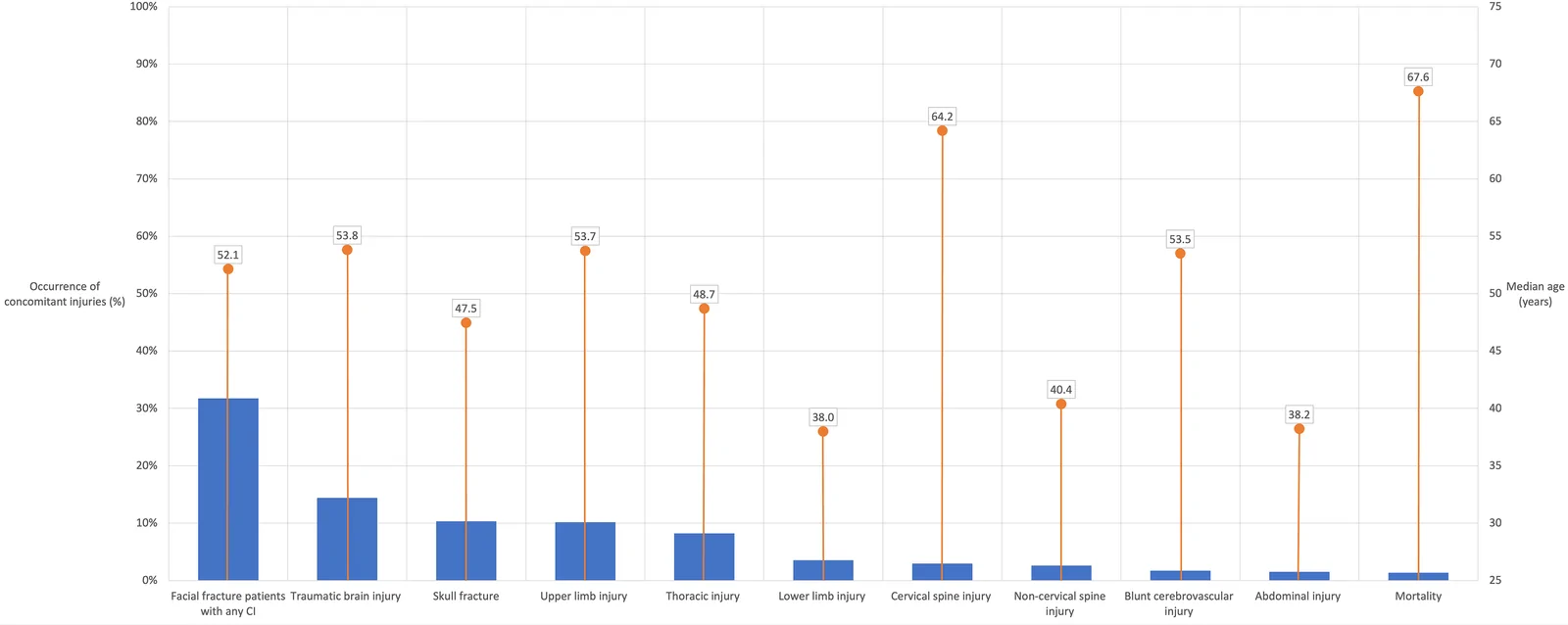
Prevalence of concomitant injuries (blue) and corresponding median age (orange)

Variation in median age was observed across types of CI and mortality (Fig. 2). The median age was 52.1 years in patients with any CI (31.7%). Among patients suffering from cervical spine injury (2.9%), the median age was as high as 64.2 years. In contrast, the median age was as low as 38.0 years among patients suffering from lower limb injury (3.5%). The median age for mortality (1.3%) was 67.6 years.

**Fig. 3:**
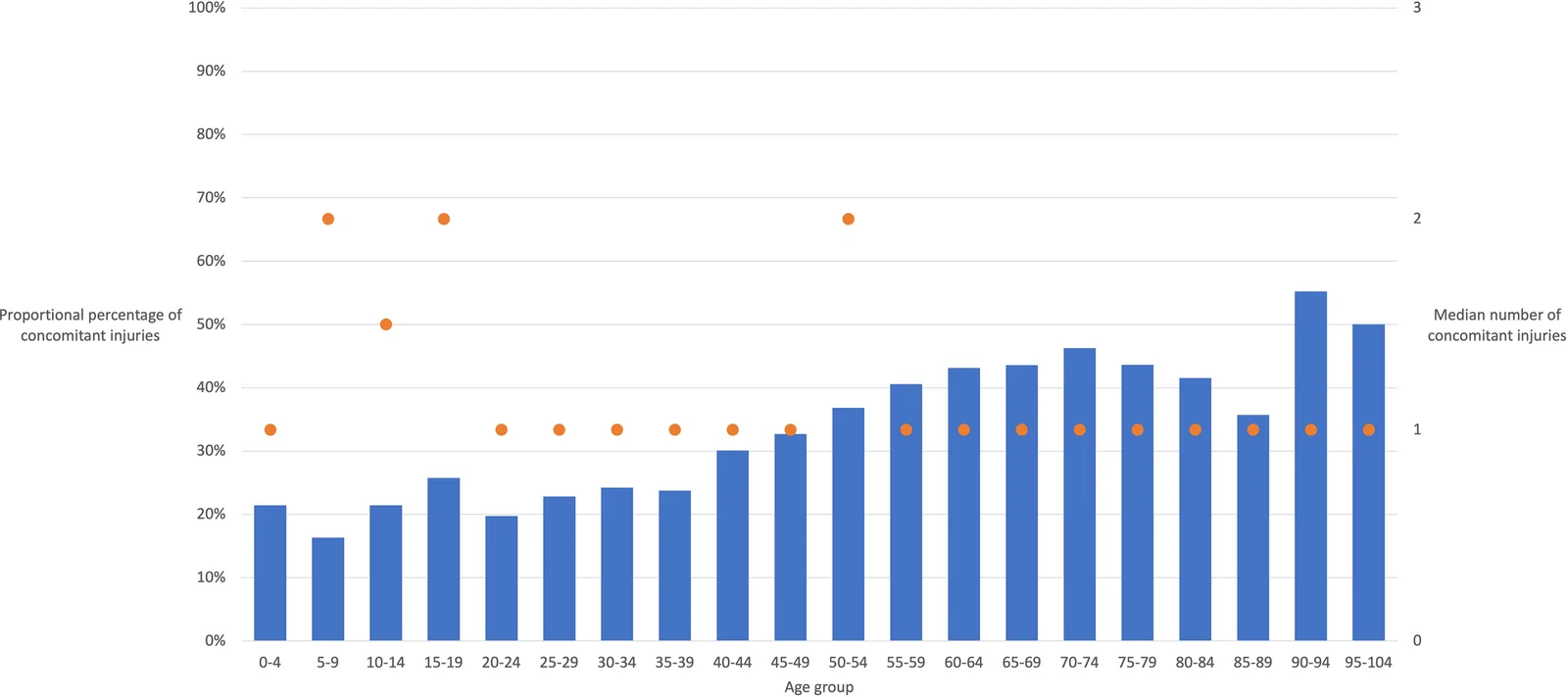
Proportional percentage (blue) and median number of concomitant injuries (orange) in different age groups,

Major differences in occurrence of CIs were observed across age groups (Fig. 3). The occurrence of CI increased gradually from 19.8% in the 20–24-year age group to over 46% in the 70–74-year age group. Among facial fracture patients in the 5–9-year age group, 16.3% suffered from CIs. In contrast, the most dominant age group was 90–94 years, where over 55% of patients suffered from CIs. Examination of median number of CIs in each age group revealed that nearly all age groups received values of one (Fig. 3). Only three of the 20 age groups received values of two, while the 10–14-year age group received a value of 1.5.

**Fig. 4:**
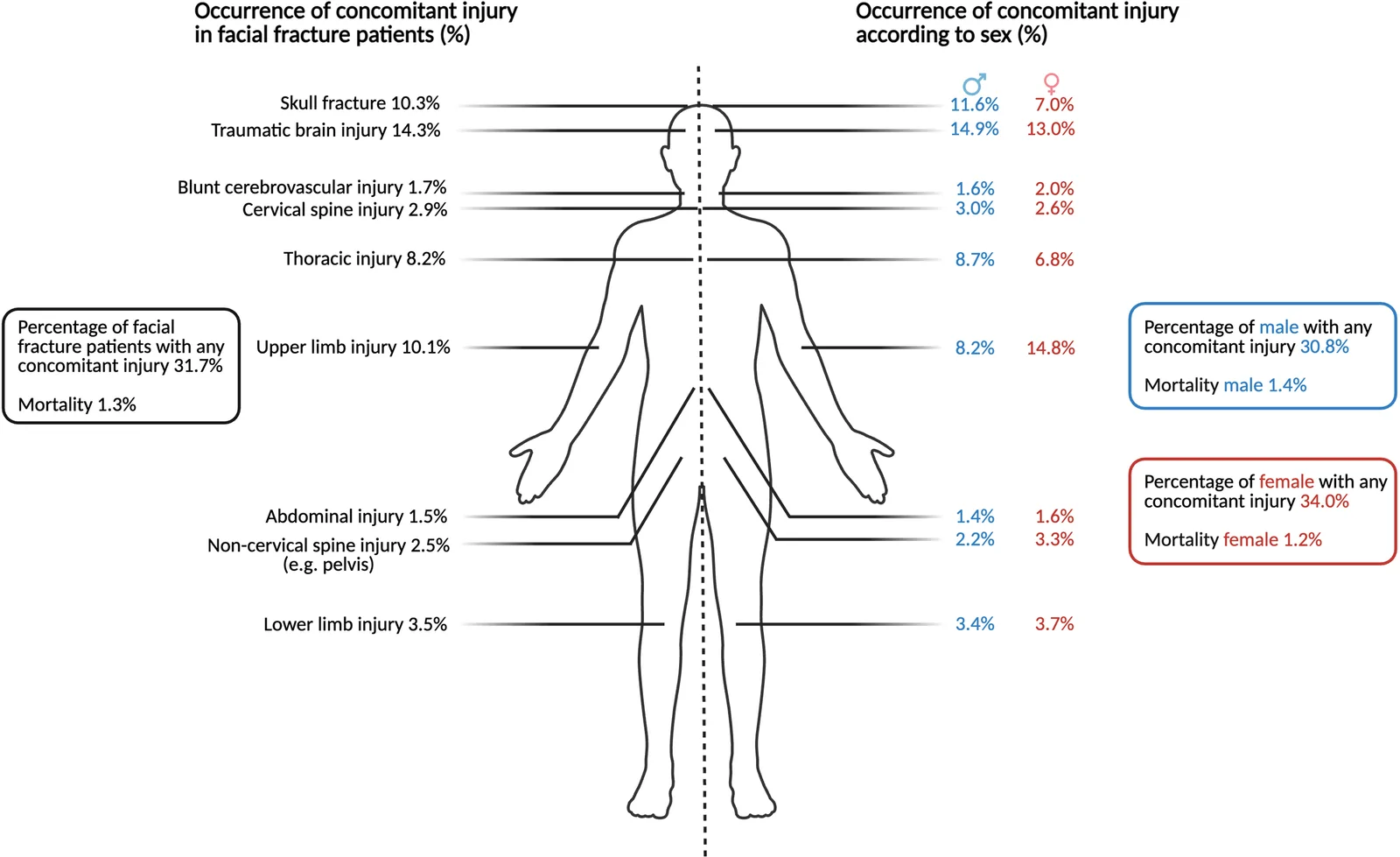
Occurrence of concomitant injury in facial fracture patients

Sex-based differences were observed in different types of CI (Fig. 4). For male patients, the predominant CI was traumatic brain injury (14.9%). In contrast, the predominant concomitant injury for female patients was upper limb injury (14.8%). In addition, male patients sustained skull fractures more frequently than female patients (11.6% vs. 7.0%).

**Fig. 5:**
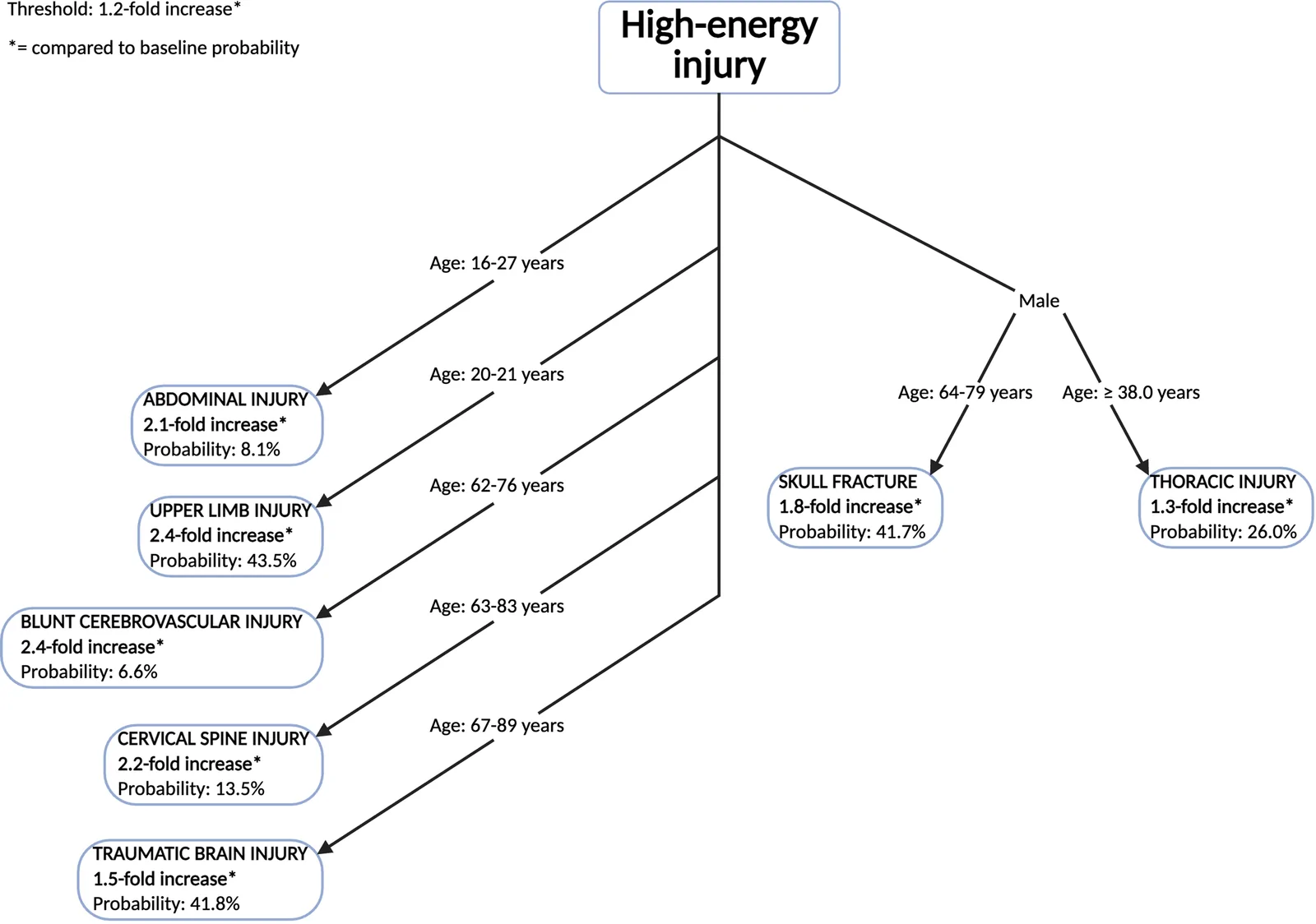
Concomitant injuries caused by high-energy injury mechanisms and the most prominent risk group for each injury type. The threshold for the risk group was set to 1.2-fold increased probability compared with the baseline probability of the whole population suffering from concomitant injury. Results were reported only for groups with at least 10 cases (*n* ≥ 10). The age group thresholds represent model output

**Fig. 6:**
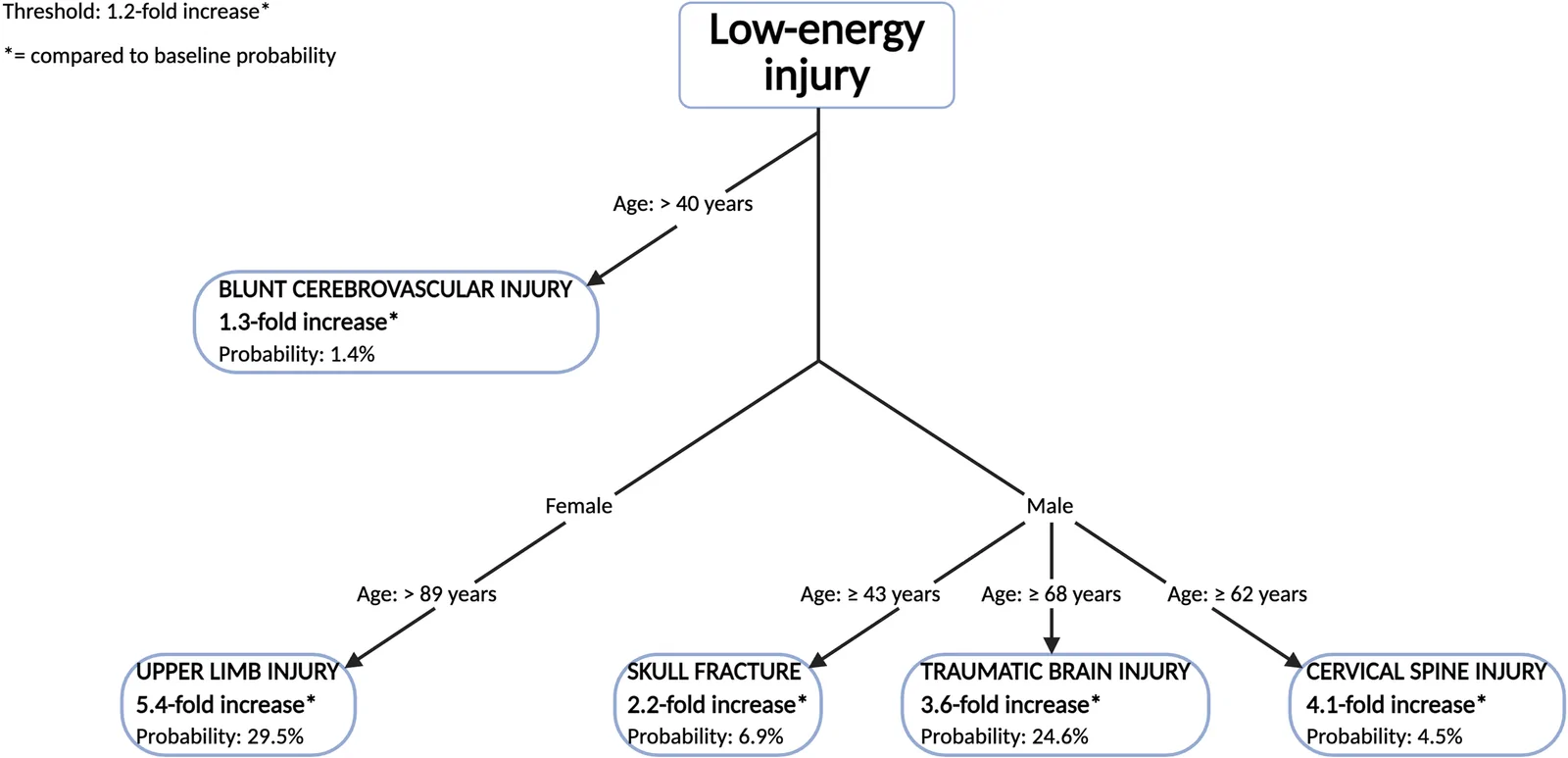
Concomitant injuries caused by low-energy injury mechanisms and the most prominent risk group for each injury type. The threshold for the risk group was set to 1.2-fold increased probability compared with the baseline probability of the whole population suffering from concomitant injury. Results were reported only for groups with at least 10 cases (*n* ≥ 10). The age group thresholds represent model output

When assessing risk groups for different types of CIs caused by high-energy injury mechanisms (Fig. 5) and low-energy injury mechanisms (Fig. 6), notable differences were observed. Detailed statistics for these exploratory risk groups, including subgroup denominators, event counts, absolute risks, and 95% confidence intervals, are provided in Table 3. Examination of high-energy injury mechanisms revealed that patients aged 19.7–21.4 years had a 43.5% probability of sustaining upper limb injuries, a 2.4-fold increase relative to the baseline probability. In contrast, examination of upper limb injuries caused by low-energy injury mechanisms revealed that females aged 89.8 years or older had a 29.5% probability of sustaining upper limb injuries, a 5.4-fold increase relative to the baseline probability.

**Table 3:** Exploratory risk groups for concomitant injuries in patients with facial fractures: subgroup denominators, event counts, absolute risks, and 95% confidence intervals (Wilson). Calculations were not performed for risk groups with a low number of observed events (indicated by a dash in the table)

High-energy injury				
Risk group	*n*	Events	Absolute risk	95% Confidence interval
Cervical spine injury Age 62.5–83.2	244	33	0.135	0.098; 0.184
Blunt cerebrovascular injury Age 62.4–76.4	198	13	0.066	0.039; 0.109
Skull fracture Males age 64.2–79.1	127	53	0.417	0.335; 0.504
Thoracic injury Males age > 38	580	151	0.26	0.226; 0.298
Abdominal injury Age 15.6–26.9	273	22	0.081	0.054; 0.119
Upper extremity injury Age 19.7–21.4	46	20	0.435	0.302; 0.578
Lower extremity injury	-	-	-	-
Traumatic brain injury Age 67.3–89.4	196	82	0.418	0.352; 0.488
Non-cervical spine injury	-	-	-	-

Low-energy injury				
Risk group	n	Events	Absolute risk	95% Confidence interval
Cervical spine injury Males age > 62	337	15	0.045	0.027; 0.072
Blunt cerebrovascular injury Age >40.6	1414	20	0.014	0.009; 0.022
Skull fracture Males age >43.2	739	51	0.069	0.053; 0.090
Thoracic injury	-	-	-	-
Abdominal injury	-	-	-	-
Upper extremity injury Females Age >89.8	61	18	0.295	0.196; 0.419
Lower extremity injury	-	-	-	-
Traumatic brain injury Males age >68.2	252	62	0.246	0.197; 0.303
Non-cervical spine injury	-	-	-	-

## Discussion

The overall occurrence of CIs in patients with facial fractures is variable. The patterns of these injuries vary markedly across sex and age groups, and neither high-energy nor low-energy mechanisms alone reliably rule out the presence of CIs. Our hypothesis was confirmed, as data mining enabled the identification of potentially underrecognized risk profiles.

The clinical relevance of these findings lies in addressing persistent diagnostic gaps, as facial injuries may distract clinicians from underlying CIs. It is important to distinguish these exploratory findings from established knowledge, such as the high risk associated with high-energy trauma mechanisms.

CI occurrence in facial fracture populations has been highlighted especially in high-energy injury mechanisms such as traffic accidents and high-energy falls [1, 3–5, 7, 9, 15, 16]. Our study results revealed that in high-energy injuries 57.5% of facial fracture patients sustained at least one CI. The corresponding proportion in low-energy injuries was 17.2%. However, most of the facial fractures occur after a low-energy injury mechanism such as ground-level falls [3, 4, 7, 9, 17]. Our results showed a similar trend; 64.0% of the injuries were classified as low-energy. It is evident that patients sustaining high-energy injuries are more often assessed according to an extensive trauma protocol. Notably, 41.1% of patients with a CI had two or more CIs. However, identifying CIs may be challenging, particularly in cases of minor injury. Regarding low-energy facial fractures, the data-driven analysis of the current study highlights the importance of a systematic evaluation, especially for upper limb and head-and-neck injuries in patients.

Although facial fractures are more common in the male population [1–3, 5, 6, 18], earlier studies have not found differences in the odds of sustaining CIs [3, 5]. Interestingly, in our study, female patients had more concomitant injuries than males (34.0% vs. 30.8%), and differences also emerged in CI types. Compared with females, males had more traumatic brain injuries (14.9% vs. 13.0%) and skull fractures (11.6% vs. 7.0%), whereas females had notably more upper limb injuries than males (14.8% vs. 8.2%). Sex-related differences in trauma mechanisms may partly explain the observed injury patterns. In particular, the male predominance in head injuries might reflect the greater role of interpersonal violence in facial fractures among men [19–21]. In addition, a study by Galteland et al. [22] showed that the majority (72%) of bicyclists sustaining head injuries were male, and there was a link between major head injuries and facial fractures.

The risk of CI associated with ageing in patients with facial fractures has been demonstrated in numerous studies [4, 7, 15, 17, 23], and the risk is particularly associated with brain injuries [4, 7, 17, 23], cervical spine injuries [4, 7], and mortality [4, 7, 23], as confirmed also by our results. As a novel contribution to the literature, we detected a new risk group of CI. Female patients who sustain facial fractures from low-energy mechanisms—especially those aged 90 years or more—often present with an upper limb injury too. The emphasis on sex is likely explained not only by the larger proportion of elderly women than men at a population level but also by their greater susceptibility to falls. Overall, women are susceptible to injuries from ground-level falls [8]. Typical characteristics of the elderly make them more prone to accidents in daily life. Elderly have, for instance, reduced eyesight and perception and deficits in motor skills and balance, predisposing them to falls [19, 24]. In addition, low-energy mechanisms have enough force to cause injuries in elderly individuals in contrast to younger people [25].

Thus, the occurrence of CIs increases progressively with age, peaking in the age group 90–94 years, where over 55% of patients experienced CIs. Mortality of facial fracture patients is greater in the geriatric population and is linked to under-triage of the elderly [4], which is an issue warranting consideration in clinical settings. However, substantial number of CIs were also present in other age groups. Of patients aged 15–19 years, nearly 30% experienced CIs, and the median number of CIs was two, as it was also in 5–9-year-olds. This is most likely explained by the notable share of high-energy injury mechanisms causing facial fractures in adolescents and young adults [26] and in children [5].

Due to the retrospective study design, some data might be missing or inadequate, especially regarding specific injury mechanisms. In addition, there is a potential risk of ascertainment bias, as patients with high-energy trauma are more likely to undergo extensive imaging than those with low-energy trauma. Imaging in this cohort followed the institutional trauma protocols of Helsinki University Hospital, which are aligned with European clinical care standards, although some temporal variation in imaging practices may have occurred over the study period. Also, the mortality rates might be underestimated since some of the patients might have been transferred to other hospitals for further treatment and rehabilitation. Furthermore, the decision-tree models focused on early clinically available variables. The exclusion of other factors, such as specific fracture locations or injury severity, limits the interpretability of these model-derived subgroups. Also, as CIs in the present study were classified based on radiological confirmation, the identified CI risk groups reflect patterns of diagnosed injuries rather than a demonstrated reduction in missed diagnoses. These results should be considered hypothesis-generating and interpreted as preliminary findings. Nevertheless, they may help identify subgroups within the facial fracture population that are at increased risk of concomitant injuries, thereby informing the design of future targeted studies.

In conclusion, diagnosis and treatment of facial fracture patients often require cooperation from a versatile multidisciplinary team [20, 27]. Due to high CI risk in facial fracture patients, awareness of the injury profiles among clinicians is essential. The risk groups identified in this study are exploratory and should not be considered validated clinical decision rules. However, these findings support the need for systematic trauma assessment. In conjunction with the necessary assessment and imaging of the facial trauma, we encourage use of the Advanced Trauma Life Support (ATLS) protocol [28] to routinely rule out CIs. The risk groups identified in this study may serve as a supplemental guide to heighten clinical suspicion for CIs, particularly in patients with low-energy injuries. On the other hand, it is important to identify patient groups with a low risk of CI who would not benefit from, for example, extensive trauma imaging.

## Supplementary Information

Below is the link to the electronic supplementary material.

Supplementary Material 1

## Data Availability

Datasets used and/or analysed in the current study are available from the corresponding author upon reasonable request as permitted by the data protection regulations of the party granting the research permit.

## Declarations

### Article suitability for European journal of trauma and emergency surgery

The study focuses on concomitant injuries of facial fracture patients. The information and the study results that we provide are applicable in a clinical setting as well as in scientific studies. The results can be used inter alia in the fields of oral and maxillofacial surgery and emergency medicine.
