# Supplementary material for: Exploring risk groups for concomitant injuries in facial fracture patients: a data-driven analysis
Source: Eur J Trauma Emerg Surg. 2026 Sep 29;52(1):303. doi: 10.1007/s00068-026-03342-x (PMC13624038; doi:10.1007/s00068-026-03342-x)
Supplement: Supplementary file 1 [file 68_2026_3342_MOESM1_ESM.docx]

**Supplementary material file 1:**

**Manuscript:** Exploring risk groups for concomitant injuries in facial fracture patients: A data-driven analysis

**Detailed description of the parameters used for creating the data-driven model**

Decision tree analysis was performed as an exploratory subgroup discovery method to identify clinically interpretable patient subgroups and age thresholds associated with specific associated injuries. This model was not developed to act as a predictive model. Separate analyses were performed for low-energy and high-energy trauma. Within each trauma energy group, individual decision trees were generated for each associated injury outcome (traumatic brain injury, skull fracture, blunt cerebrovascular injury, cervical spine injury, upper extremity injury, lower extremity injury, thoracic injury, spinal injury, and abdominal injury).

The candidate predictor variables were age, sex, and injury mechanism. Decision trees were constructed in Python (version 3.14.2) using the DecisionTreeClassifier implementation in scikit-learn (version 1.51.1). Node splitting was based on the Gini impurity criterion. To maintain clinical interpretability and avoid overly complex subgrouping, tree depth was limited to three levels (max_depth=3). This was to prioritize clinical interpretability and to avoid over-fragmentation of the study population into very small subgroups. Default scikit-learn settings were otherwise used, including a minimum of two samples required to split an internal node (min_samples_split=2) and a minimum of one sample per terminal leaf (min_samples_leaf=1). No cost-complexity pruning was applied (ccp_alpha=0.0), and no specific adjustment for class imbalance was performed (class_weight=None). A fixed random seed (random_state=42) was used to ensure reproducibility.

As the objective of the analysis was exploratory subgroup discovery rather than predictive modelling, no train/test split, cross-validation, or bootstrapping was performed. The resulting decision rules and age thresholds should therefore be interpreted as hypothesis-generating and intended to support identification of clinically relevant patient subgroups, rather than as externally validated prediction models.
